# CULTIVATING RESILIENCE ACROSS DIVERSE AGING COMMUNITIES

**DOI:** 10.1093/geroni/igae098.1727

**Published:** 2024-12-31

**Authors:** Catherine García, Heather Farmer

**Affiliations:** Chair; Co-Chair

## Abstract

This symposium explores the multifaceted experiences of resilience and well-being among older adults from diverse cultural backgrounds, emphasizing the role of resistance and assertion of cultural practices for optimal aging and aging in place. Presentations span various communities in the U.S. that are not widely studied in gerontological research, offering a comprehensive perspective on aging challenges and strategies. The first presentation focuses on the concept of ‘Eldership’ among Alaskan Native Elders in promoting mutually beneficial relations within youth, communities, tribes, and organizations. The second presentation highlights perspectives from Native Hawaiian kumu loea (expert teachers) of traditional practices on regeneration of cultural practices and language, emphasizing the challenges and personal resilience of those reclaiming indigenous knowledge through resistance. The third presentation explores the well-being of older South Asian American women, identifying factors like robust social networks, participation in cultural events, and mindfulness practices as asset-based strengths that empower and support a sense of purpose. The fourth presentation identifies factors influencing life satisfaction among resettled Bhutanese older adults, uncovering the importance of social support and resilience. The final presentation investigates self-employment among older Hispanics, revealing intragroup disparities in safety-net program access and economic opportunities, showcasing how cultural identity serve as a proxy to later life financial security and well-being. Together, these presentations foster a deeper understanding of resilience across diverse aging communities, emphasizing the importance of cultural resistance and traditional practices as integral components of well-being, providing insights for those seeking culturally sensitive approaches to support the well-being of older populations.

